# Intracavitary/Interstitial Applicator Plus Distal Parametrial Free Needle Interstitial Brachytherapy in Locally Advanced Cervical Cancer: A Dosimetric Study

**DOI:** 10.3389/fonc.2020.621347

**Published:** 2021-02-18

**Authors:** Hong-Da Qu, Dong-Mei Han, Ning Zhang, Zhuang Mao, Guang-Hui Cheng

**Affiliations:** Department of Radiation Oncology, China–Japan Union Hospital of Jilin University, Changchun, China

**Keywords:** cervical cancer, interstitial brachytherapy, dosimetric analysis, treatment outcome, parametrial interstitial brachytherapy

## Abstract

**Purpose:**

To explore the dosimetric advantage of combining intracavitary/interstitial applicator with distal parametrial free needle interstitial brachytherapy (IC/IS+ISBT DP) based on MRI for locally advanced cervical cancer.

**Methods and Materials:**

77 IC/IS+ISBT DP treatment plans were developed for 34 patients with locally advanced cervical cancer from June 2016 to January 2020 in this study. We removed the free needles and devised a new IC/ISBT treatment plan based on the same principle. We then compared the dosimetric differences of D90, D98, V100, V150, V200 for HR-CTV (high-risk clinical target volume), D90 for IR-CTV (Intermediate risk-CTV) and D2cc for OARs (organs at risk) between the two groups of treatment plans for the same patient, and the paired T test was performed in parallel. Further, the dosage differences between the two group plans under different parametrial extension widths (the maximum distance of HR-CTV from the vertical direction of the uterine tandem at coronal position) were compared. The survival rate was calculated using the Kaplan-Meier method. Prognostic factors for overall survival (OS) and progression-free survival (PFS) were determined by Cox regression method. RTOG/EORTC criteria were used to grade toxicities.

**Results:**

A total of 297 free needles were used, with a weight ratio of 15.8% ± 0.11, and a mean insertion depth of 6.52cm ± 2.8cm. D90, D98, V100 for HR-CTV, and D90 for IR-CTV for IC/IS+ISBT DP were significantly higher than IC/ISBT for which free needles were removed (p<0.05). And the V200 for HR-CTV and D2cc for bladder, rectum and sigmoid were decreased (p<0.05). When the parametrial extension widths were greater than 3cm, the HR-CTV D90 and the D2CC for rectum, bladder and sigmoid colon for IC/IS-ISBT DP were advantageous compared to IC/ISBT (p<0.05). The 2-yr OS, PFS and local control rate (LC) were 82.3, 66.8, and 93.1%, respectively. Parametrial extension widths was the only statistically prognostic factors for PFS (p = 0.002) on univariate analysis. No grade 3 or 4 Treatment-related toxicities were observed.

**Conclusion:**

Our institutional experiences showed that IC/IS+ISBT DP is an effective treatment for cervical cancer patients with distal parametrial extension. IC/IS-ISBT DP had dosage advantage and clinical feasibility in locally advanced cervical cancer with distal parametrial extension when the parametrial extension widths were greater than 3cm.

## Introduction

The combination of external beam radiotherapy (EBRT), concurrent chemotherapy and brachytherapy (BT) is the standard treatment for locally advanced cervical cancer, among which brachytherapy is indispensable (1, 2). Brachytherapy can be divided into intracavity (ICBT), interstitial (ISBT) and combined intracavitary/interstitial brachytherapy (IC/ISBT). ICBT has been used for decades and is most suitable for small-volume and superficial lesions. However, when the tumor is bulky, eccentrically located or irregular in shape, problems can arise such as poor target volume coverage, insufficient dose, or excessive radiation to organs at risk (OAR). To avoid these potential problems, IC/ISBT was developed, which can increase the fitness of target volume and the uniformity of dose distribution (3). Nevertheless, the dose coverage of IC/ISBT in cervical cancer with distal parametrial extension remained limited (4). For improved parametrial coverage, free needles have been implanted into distal parametrial tumor extensions in combination with IC/ISBT. However, the dosimetry of this combined technique has rarely been reported. The purpose of this study was to investigate the dosimetric advantages of IC/IS+ISBT DP for locally advanced cervical cancer with distal parametrial extension, and to report the result of this radiation technique.

## Materials and Methods

### Patient Selection and Treatment

The study comprised 34 patients with locally advanced cervical carcinoma, for whom 77 IC/IS+ISBT DP treatment plans were developed, starting in June 2016 and extending to January 2020. The average age was 53 years (range, 36–76 years), with a median age of 54 years. The pathology diagnoses included 32 cases of squamous cell carcinoma, and 2 cases of adenocarcinoma. Disease staging for patients admitted prior to September 2018 was based on the 2009 FIGO system, and patients admitted after September 2018 were based on the 2018 FIGO system. All patients were free of distant metastasis prior to treatment. And 7 patients had received 1-3 cycles of neoadjuvant chemotherapy (TP), but still inoperable, were transferred to our department to receive definitive radio(chemo)therapy. All patients were treated with EBRT (45 Gy in 25 fractions), administered *via* intensity modulated radiation therapy (IMRT) or three dimensional conformal radiotherapy (3D-CRT). Patients with pathology or imaging-positive lymph nodes received an increased dose of 60 Gy. Platinum-based single-drug or dual-drug concurrent chemotherapy was administered during the EBRT. Single-agent cisplatin was administered at a dose of 30–40 mg/m^2^ once a week for 5 weeks. Dual-drug concurrent chemotherapy (60 mg/m^2^ cisplatin + 60 mg/m^2^ docetaxel or 135 mg/m^2^ paclitaxel every three weeks for 3 cycles) was considered for patients with large-volume tumor in order to control micrometastasis as much as possible, although those patients who could not tolerate chemotherapy were treated with radiotherapy alone.

Gynecologic physical examination and pelvic MRI were performed within one week prior to initiating brachytherapy. The appropriate applicator was selected according to the patient’s tumor regression. The 77 treatment plans used IC/IS+ISBT DP to include as much of the distal parametrial extension as possible.

### Applicator Implantation Method and Dose Calculation

MRI-guided high-dose-rate (HDR) 192Ir BT (Micro-Selectron HDR) was performed for all patients.

The implantation process proceeded according to the following description. Prior to the operation, different angles of the uterine tandem were chosen according to the position of the uterus. The tumor location, size, shape, parametrial invasion and the relationship with the surrounding organs were derived from the MRI images before and after EBRT, as well as the gynecological examination. These guided the selection of the appropriate needle position, implantation depth, the depth from the perineum to the tumor, and the route of needle insertion. Preoperatively, the patient underwent vaginal irrigation, urinary indwelling catheterization and enema preparation. Under general anesthesia, the applicator (Utrecht/circular applicator) was implanted under real-time ultrasound guidance in accordance with the preoperative preset; the depth of the needle in the insertion points could be adjusted utilizing ultrasound guidance. Evaluate the distance of HR-CTV from the lateral edge of the parametrium to the needles in the fixed insertion points combined with ultrasound on transverse position. If the distance exceeds 1.3–1.5cm, carefully consider whether to increase the free needles on distal parametrium (the Near Maximum Distance approximately exceed 3–3.2cm). Upon completion of the implantation, with needles fixed, the rectal pressure plate was used to push open the rectum. The prescription dose delivered was 7 Gy × 4 f, 1 week/fraction.

Magnetic resonance images were obtained by 3.0 T MRI scanner after the implantation was completed. Images include sagittal, axial and coronal T2-weighted turbo spin-echo images, with a slice thickness of 4.5 mm and a balanced steady-state free precession scan with 1.5 mm slice thickness for applicator reconstruction purposes.

### Contouring and Treatment Planning

HR-CTV and IR-CTV were contoured based on MRI and clinical examination before and after EBRT, according to the recommendation of the GEC⁃ESTRO (5). Contoured OARs included the bladder, rectum, sigmoid and small bowel. We contour the HR-CTV as the entire cervix and any notable remaining tumor at BT (5). Specific borders for HR-CTV were delineated according to the recommendation of Viswanathan et al. (6). In general, the lower border covered the inferior cervical margin. For disease that extended to the vagina, the lower border was determined through clinical examination. Superiorly, the HR-CTV was contoured at the level where the uterine cavity appeared, further extending 1cm upwards to form a cone-shaped tip. To encompass intrauterine disease, the HR-CTV was defined by sagittal T2-weighted MRI images. Any lateral parametrial disease extension required contouring based on the combination of clinical gynecologic examination and MRI images. The IR-CTV represents the initial gross tumor volume (GTVinit) as superimposed on the topography at the time of brachytherapy, together with a margin surrounding the HR-CTV in areas without an initial GTVinit. By definition, the IR-CTV includes all of the HR-CTV and safety margins as appropriate.

### Plan Design and Evaluation

Under the guidance of MRI, the applicator and the implant needles were reconstructed in the treatment planning system (TPS), and the source dwell point of the radioactive source was selected according to the shape of the target area and the relative, three dimensional positional relationship of the OARs. We optimized the treatment plan based on the prescribed dose and OARs dose limits. The final objective was a total dose (combined EBRT and BT) of HR-CTV D90 ≥ 85 Gy, IR-CTV D90 ≥ 60 Gy, bladder D2cc ≤ 80 Gy, and rectum/sigmoid/small bowel D2cc ≤ 70 Gy. Using the linear-quadratic (LQ) model to calculate the biologically equivalent dose in 2 Gy fractions (EQD2), normal tissue α/β=3, tumor α/β=10, the formula is as follows (7):

$$EQD2=\frac{Nd\left(1+\frac{d}{\alpha /\beta }\right)}{1+\frac{2}{\alpha /\beta }}$$

Each fractional treatment plan of IC/IS+ISBT DP group generated a simulation plan leaving the free needles, defined as the IC/ISBT research group. However, this optimized IC research plan was not used clinically. The aim dose to be delivered to the target and the limit dose to the OAR in the IC/ISBT research plan were the same as the criteria of the IC/IS+ISBT DP group.

### Follow-Up

All patients were clinically evaluated by imaging studies (MRI or CT scan) and bimanual pelvic examination at the first 1 month and once every three months during the first 2 years after the completion of all treatments, and every 6 months thereafter. The OS time was calculated from the date of treatment start to the date of death or last follow up. The PFS time was calculated from the date of starting treatment to the date of disease progression, relapse, disease-related death, or the last follow-up. The LC time was calculated from the date of starting treatment to the date of local recurrence or last follow up.

CR were defined as a 100% decrease in gross tumors. And we defined partial response (PR) as ≥ 50% decrease of primary gross tumor. Treatment response was evaluated by imaging studies (MRI or CT scan) and bimanual pelvic examination at 3 months after completion of brachytherapy. RTOG/EORTC criteria are used to grade toxicities (8).

### Data Processing and Statistical Analysis

**Figure 1:**
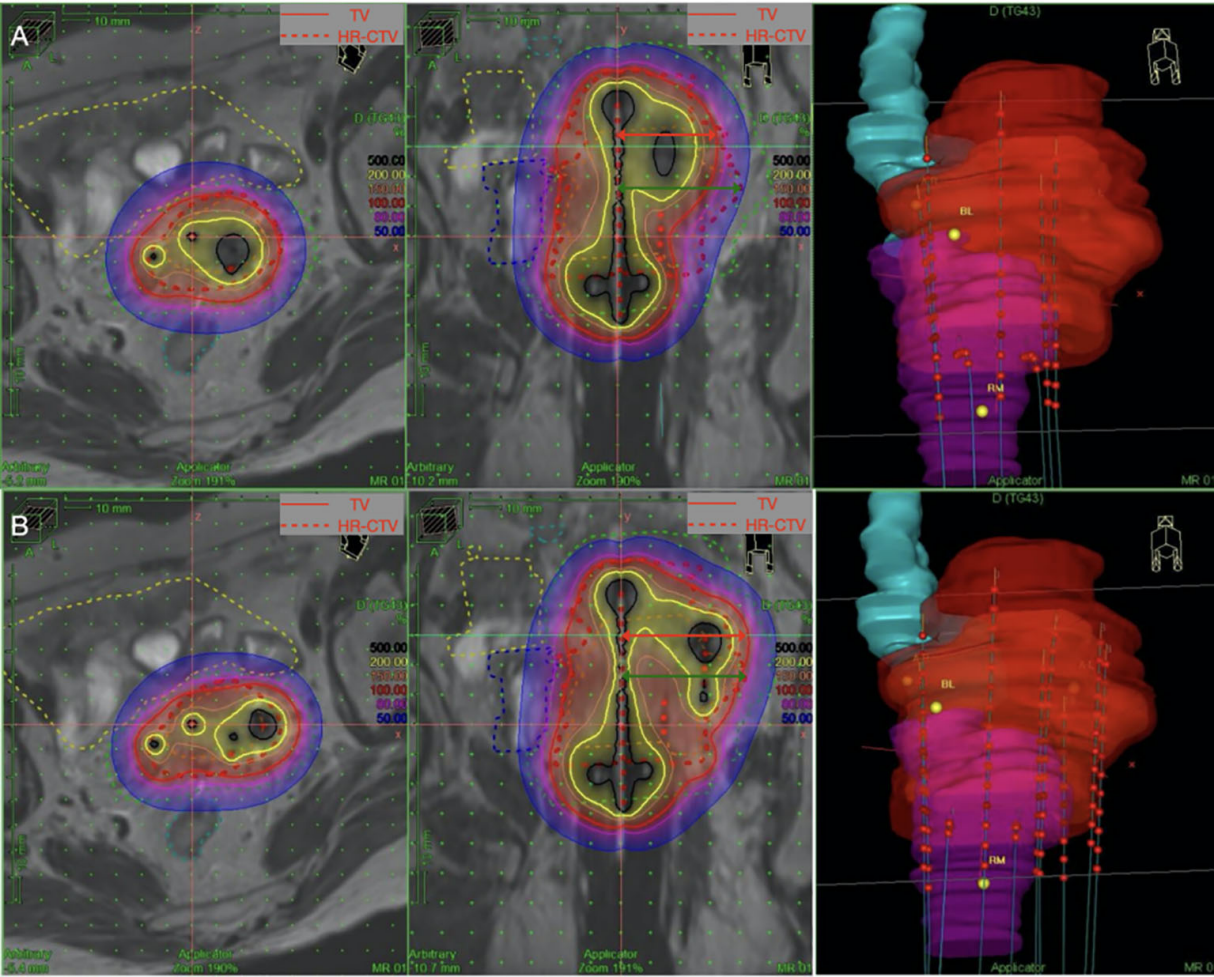
NMD (red arrow) is defined as the maximum distance of the 100% isodose curve from the vertical direction of the uterine tandem at coronal position. Parametrial extension widths (green arrow) is defined as the maximum distance of HR-CTV from the vertical direction of the uterine tandem at coronal position. The parametrial extension widths of this patient is 4.06cm. **(A)** The standard loading of the IC/ISBT for which free needles were removed shows insufficient coverage of the HR-CTV (D90: 6.06Gy, V100: 83.21%,NMD:3.18cm). **(B)** Loading of the free needles on the left improves the coverage of the HR-CTV(D90:7.15Gy,V100:91.66%,NMD:4.19cm). CTV, clinical target volume; HR, high risk; D90, the minimum dose delivered to 90% of the target volume. TV, treatment volume, target area covered by the 100% isodose curve.

The height, width, thickness, volume, and the near maximum distance from uterine tandem (NMD, **Figure 1**) of the treatment volume (TV, target area covered by the 100% isodose curve) between the two groups was compared using paired t-test. Likewise, the paired t-test was performed on the HR-CTV D90, D98, V100, V150, V200, IR-CTV D90, and OAR D2cc of these two groups before and after. Furthermore, we compared dosimetric differences for the different parametrial extension widths between these two groups. OS, PFS and LC were calculated using the Kaplan–Meier method. Potential prognostic factors for OS and PFS were investigated by using univariate Cox regression models. Differences were considered significant at P<0.05 (2-sided). All statistical analyses were performed using GraphPad Prism 8 Software (GraphPad Software, Inc, San Diego, CA).

## Results

### Patient Characteristics

All 34 patients completed EBRT with 45 Gy/25 f and received brachytherapy 28Gy/4f in our department. Characteristics for 34 patients are shown in **Table 1**.

**Table 1:** Patient characteristics and treatment parameters.

Characteristic	Value
Age at primary diagnosis (years)	Median:54 (range: 36–76)
Tumor stage (FIGO2009)	
IIB	9
IIIA	2
IIIB	10
** Ⅳ**A	1
Tumor stage (FIGO2018)	
IIB	1
IIIc1	7
IIIB	3
** Ⅳ**A	1
Neoadjuvant chemotherapy	
TP	7
none	27
Concurrent chemotherapy	
TP	1
DP	6
Single-drug platinum	18
none	9
Sequential chemotherapy	
TP	7
none	27
Follow up (day)	Median: 710 (241–1,696)
HR-CTV D90 (Gy)	91.15 ± 4.06
IR-CTV D90 (Gy)	66.64 ± 3.74
Bladder D2cc (Gy)	78.88 ± 6.02
Rectem D2cc (Gy)	67.31 ± 5.25
Sigmoid D2cc (Gy)	64.30 ± 6.35
small bowel D2cc (Gy)	62.02 ± 6.30
Treatment outcome	
CR	18
PR	16

FIGO, International Federation of Gynecology and Obstetrics.

TP, cisplatin plus paclitaxel; DP, Cisplatin plus docetaxel.

CTV, clinical target volume; HR, high risk; IR, intermediate risk; D90, the minimum dose delivered to 90% of the target volume; D2cc, minimal dose to 2 cm^3^.

CR, complete remission; PR, partial response.

### Characteristics of Brachytherapy

A total of 77 IC/IS+ISBT DP fractions were administered to 34 patients using 1054 needles, including 297 free needles. The mean number of free needles was 3.86 (range, 1–9). The weight ratio of these free needles was 15.8% ± 0.11, and the mean insertion depth was 6.52cm ± 2.8cm.

### Volumetric Comparison

We compared the volumetric of the IC/IS+ISBT DP treatment plan with the IC/ISBT treatment plan for which the free needles had been removed. The comparison results of the height, width, thickness, volume and NMD of TV between the two treatment plans are shown in **Table 2**. The width, NMD and volume of the TV IC/IS+ISBT DP group are significantly greater than the IC/ISBT group, while the thickness was slightly lower (*p*<0.05).

**Table 2:** Comparison of mean values of height, width, thickness, volume and NMD between TV_IC/IS+ISBT DP_ and TV_IC/ISBT_ (n=77).

Parameter	TV_IC/IS+ISBT DP_	TV_IC/ISBT_	P^*^
**Height (cm)**	7.14	7.10	0.494
**Width (cm)**	6.0	5.23	<0.001
**Thickness (cm)**	3.71	3.82	0.027
**NMD (cm)**	3.64	2.90	<0.001
**Volume (cc)**	47.54	45.76	<0.001

NMD, the near maximum distance from uterine tandem; TV, treatment volume, target area covered by the 100% isodose curve.

^*^Matched pairwise two tailed t-test statistics.

### DVH Parameters

The comparison results of the dose-volume histogram (DVH) parameters of these two treatment plans are listed in **Table 3**. Notably, the D90, D98, V100 of HR-CTV and D90 of IR-CTV for the IC/IS+ISBT DP group were significantly higher than that of the IC/ISBT group, while the HR-CTV V200 was slightly lower (*p*<0.05). Regarding the protection of OARs, all treatment plans met the single dose limit (bladder D2cc<5.88 Gy; rectum, sigmoid colon and small intestine D2cc<4.98 Gy). Importantly, the D2cc of the bladder, rectum and sigmoid of IC/IS+ISBT DP were lower than IC/ISBT (p<0.05).

**Table 3:** Comparison of dose distribution between IC/IS+ISBT DP and IC/ISBT (Gy, α±s).

Physical dose	IC/IS+ISBT DP (A)	IC/ISBT (B)	A-B	P^*^
HR-CTV				
D90	8.11 ± 0.52	7.83 ± 0.70	0.27 ± 0.38	<0.001
D98	6.79 ± 0.56	6.29 ± 0.84	0.50 ± 0.53	<0.001
V100	0.966 ± 0.036	0.949 ± 0.036	0.017 ± 0.037	<0.001
V150	0.650 ± 0.067	0.652 ± 0.064	−0.002 ± 0.043	0.686
V200	0.370 ± 0.069	0.394 ± 0.064	−0.024 ± 0.042	<0.001
IR-CTV				
D90	4.79 ± 0.46	4.35 ± 0.64	0.44 ± 0.44	<0.001
Rectem D2cc	4.15 ± 0.56	4.38 ± 0.77	−0.23 ± 0.44	<0.001
Bladder D2cc	5.46 ± 0.50	5.56 ± 0.59	−0.1 ± 0.30	0.007
Sigmoid D2cc	3.78 ± 0.83	3.90 ± 0.94	−0.12 ± 0.30	<0.001
small bowel D2cc	3.55 ± 0.91	3.57 ± 0.92	−0.02 ± 0.18	0.161

CTV, clinical target volume; HR, high risk; IR, intermediate risk; D90 and D98, the minimum dose delivered to 90 and 98% of the target volume, respectively. V100, V150, and V200, percent volume of the target receiving at least

100, 150, and 200% of the prescribed dose, respectively. D2cc, minimal dose to 2 cm^3^.

^*^Matched pairwise two tailed t-test statistics.

### Dosimetric Comparison Under Different Parametrial Extension Widths

All 77 treatment plans were divided into 4 groups according to the parametrial extension widths of ≤3, 3.1–3.5, 3.6–4.0, and >4cm, and compared the HR-CTV D90 of IC/IS+ISBT DP to the IC/ISBT treatment plans (**Table 4**).The HR-CTV D90 of IC/IS+ISBT DP was significantly higher than the IC/ISBT treatment plan for parametrial extension widths exceeding 3cm (*p*<0.05). Furthermore, we divided all treatment plans into 2 groups based on the parametrial extension width of ≤3cm, and performed paired t-tests on the D_2CC_ of the relevant OAR between IC/IS+ISBT DP and IC/ISBT treatment plans (**Table 5**). Although the D_2CC_ of bladder and small bowel showed no significant difference, the D_2CC_ of rectum, bladder, and sigmoid for IC/IS+ISBT DP showed dosimetry advantage for parametrial extension widths exceeding 3cm (*p*<0.05).

**Table 4:** Comparison of HR-CTV D90 with different parametrial extension widths (α) between IC/IS+ISBT DP and IC/ISBT (n=77).

	α≤3cm (n=35)	3cm<α≤3.5cm (n=17)	3.5cm<α≤4cm (n=15)	α>4cm (n=10)
**IC/ISBT D90 (HRCTV)**	8.16 ± 0.46 Gy	7.62 ± 0.57 Gy	7.50 ± 0.72 Gy	7.58 ± 1.09 Gy
**IC/IS+ISBT DP D90 (HRCTV)**	8.20 ± 0.46 Gy	7.96 ± 0.49 Gy	7.98 ± 0.42 Gy	8.25 ± 0.79 Gy
P^*^	0.054	<0.001	<0.001	0.002

CTV, clinical target volume; HR, high risk; D90, the minimum dose delivered to 90% of the target volume.

^*^Matched pairwise two tailed t-test statistics.

**Table 5:** Comparison of D_2CC_ for OARs with different parametrial extension widths (>3cm and ≤3cm) between IC/IS+ISBT DP and IC/ISBT (Gy, α±s).

	IC/IS+ISBT DP (A)	IC/ISBT (B)	P^*^
**Bladder D_2cc_**			
≤3cm	5.25 ± 0.51	5.30 ± 0.52	0.095
>3cm	5.64 ± 0.44	5.78 ± 0.57	0.028
**Rectem D_2cc_**			
≤3cm	3.89 ± 0.57	4.02 ± 0.71	0.027
>3cm	4.37 ± 0.45	4.69 ± 0.70	<0.001
**Sigmoid D_2cc_**			
≤3cm	3.72 ± 0.74	3.77 ± 0.75	0.020
>3cm	3.83 ± 0.92	4.00 ± 1.07	0.007
**small bowel D_2cc_**			
≤3cm	3.45 ± 0.99	3.45 ± 0.98	0.907
>3cm	3.64 ± 0.86	3.66 ± 0.87	0.476

D2cc, minimal dose to 2 cm^3^.

^*^Matched pairwise two tailed t-test statistics.

### Treatment Outcomes

**Figure 2:**
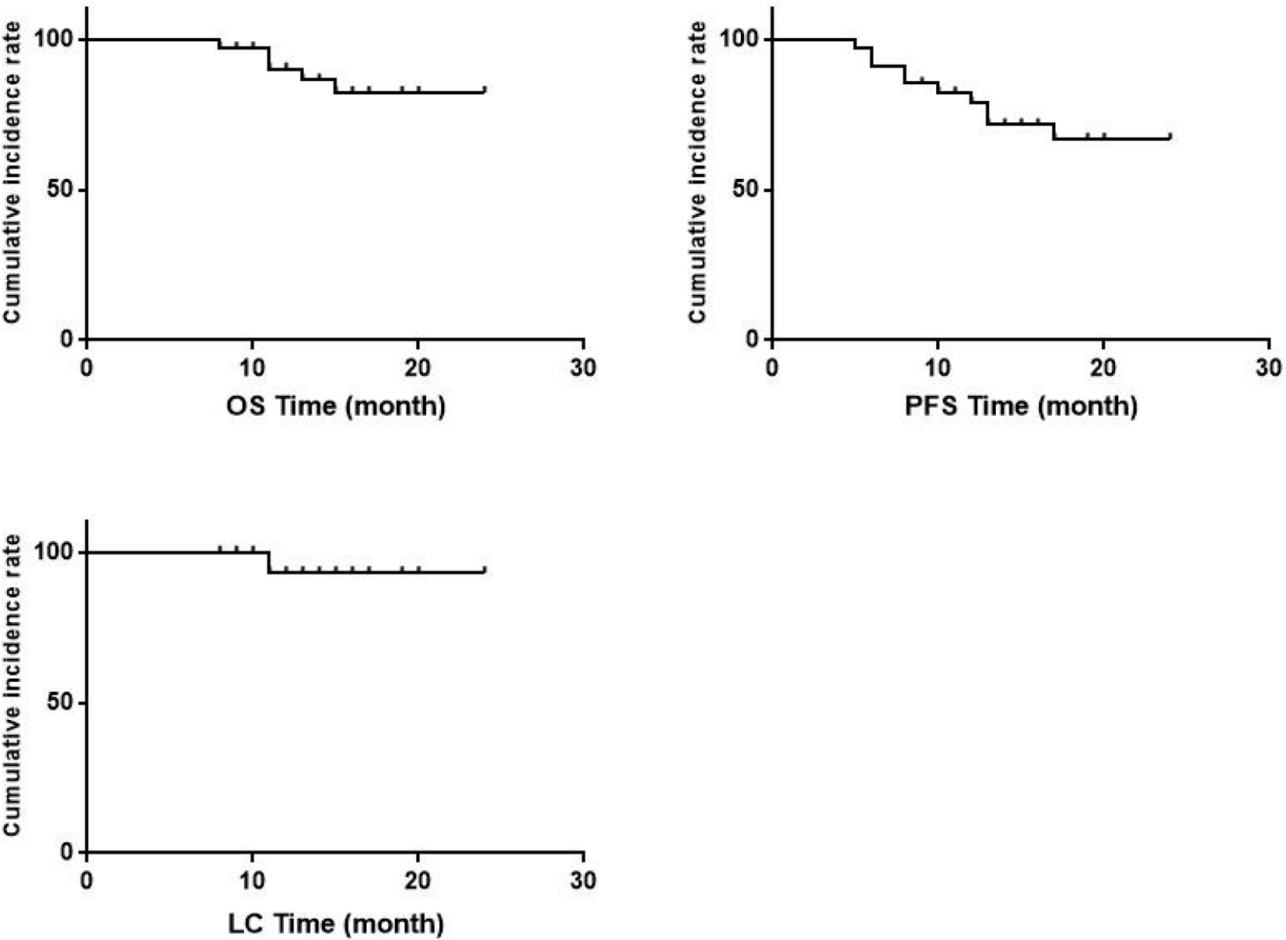
Curves of 2-yr overall survival (OS), 2-yr progression-free survival (PFS) and 2-yr local control rate (LC). No. At Risk parametrial extension widths <3cm 17 16 16 16 parametrial extension widths ≥3cm 17 8 8 8.

**Figure 3:**
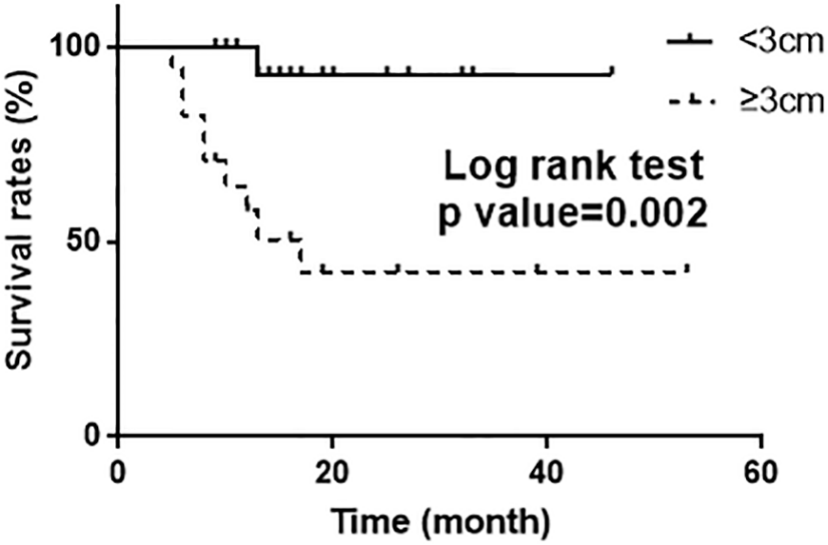
Kaplan–Meier curve of PFS (log rank test p =0.002) according to the parametrial extension widths.

Eighteen patients (52.9%) had CR and 16 patients (47.1%) had PR. The median follow-up period for 34 patients was 710 days (range, 241–1696) with 9 cases<1 year; 11 cases≥1 year, <2 years; 7 cases≥2 years, <3 year; 7 cases≥3 years. Patients received a median of 91.15GyEQD2 for D90 HR-CTV. The 2-yr OS, PFS and local control rate (LC) were 82.3%,66.8% and 93.1%, respectively (**Figure 2**). Treatment failure were experienced in nine patients during the follow-up: of which 2 patients had local recurrence and 7 had distant metastasis. In univariate analysis (**Table 6**), parametrial extension widths(<3 vs.≥3cm, p = 0.002) showed a significant effect on PFS (**Figure 3**).

**Table 6:** Prognostic factors on OS and PFS by Cox regression method.

Variables	Overall survival		Progression-free survival	
	Univariate analysis		Univariate analysis	
	HR(95%CI)	p	HR(95%CI)	p
age(<50 vs.≥50)	0.41(0.08–2.18)	0.297	1.2 (0.324–4.441)	0.785
Tumor width(<5 vs.≥5cm)	1.032(0.20–5.22)	0.969	0.285 (0.080–1.011)	0.052
Parametrial extension widths(<3 vs.≥3cm)	0.449(0.089–2.26)	0.331	0.14 (0.039–0.499)	0.002
HR-CTV D90(<90 vs.≥90Gy)	1.275(0.151–10.936)	0.834	2.443 (0.324–19.568_	0.412
Treatment response(CR vs.PR)	0.461(0.092–2.315)	0.347	0.566 (0.161–1.993)	0.375
Concurrent chemotherapy (no vs. Yes)	2.337 (0.311–17.59)	0.410	1.001 (0.212–4.713)	0.999

CTV, clinical target volume; HR, high risk; D90, the minimum dose delivered to 90% of the target volume.

CR, complete remission, PR, partial response,

HR, hazard ratio, CI, confidence interval.

### Treatment-Related Toxicities

Complications were limited to 2 cases of local infection during implantation, and were treated successfully with a single course of antibiotics. No serious bleeding or transfusions requirements occurred during implantation. There was no grade 3 or 4 late treatment-related toxicity (**Table 7**).

**Table 7:** Treatment-related toxicity.

Late toxicity	Value (n = 34)
Bladder	
Grade 0–1	30
Grade 2	4
Rectum	
Grade 0–1	32
Grade 2	2

## Discussion

Since its inception, brachytherapy has played a critical role in the treatment of cervical cancer (9). For patients with parametrial invasion, enhancements in treatment have included IC/IS applicators such as the Vienna applicator (uterine tandem + ring) and the Utrecht applicator (uterine tandem + ovoid), with IS needles implanted obliquely to the intrauterine tandem, facilitating good target coverage and resolving the problem of insufficient dose for parametrial diseases (4, 10). But the insertion point and planting direction of IS needles were limited by a fixed pinhole position and angle (4). Nomden (10) found that when distal parametrial disease extension was encountered, the HR-CTV could not be completely covered with the IC/IS applicator. Fokdal et al. (11) addressed this distal parametrial disease by the addition of free needles combined with the IC/IS applicator to cover the HR-CTV as much as possible. Because there are few relevant literature reports, the safety and dosimetry of this technology have not been clarified in detail, but it appears to be a clinically feasible treatment option. All 77 brachytherapy fractions enrolled in this study adopted IC/IS+ISBT DP. To investigate whether this technology has a dosimetry advantage over traditional IC/ISBT, we generated a simulation plan leaving the free needles, and compared it to the original plan. The comparison was limited to single treatment fractions, however, because not all of the brachytherapy fractions of the enrolled patients necessitated the free needles for distal parametrial disease extension. In most patients, free needle insertion was performed to cover the distal parametrial disease extension in the first 1–2 fractions, but owing to tumor shrinkage, conventional IC/ISBT satisfactorily covered the remaining disease. The treatment outcome demonstrated that the addition of free needles to treat distal parametrial disease could significantly improve the coverage area by TV (width, NMD and volume were all significantly increased). Specifically, the contribution to NMD was the most significant, providing an additional treatment width of about 7mm, which affected the conformity and dose to the HR-CTV. In contrast, if these needles were to be removed, in order to adequately cover the distal parametrial disease area adequately, the thickness of the treatment volume would need to be increased, which would increase the dose delivered to the bladder and rectum, as confirmed by comparison of the relevant dosimetry parameters of the two plans.

There are no reports detailing the optimal application of IC/IS+ISBT DP. Yoshida et al. (12) explored brachytherapy for different tumor volumes, modeling tumor volumes of 8 groups: 8, 12, 27, 36, 64, 80, 96, and 112cc, managed with four modalities: ICBT, IC/ISBT and ISBT. The results indicated that the HR-CTV for ISBT and IC/ISBT was better than traditional ICBT when the tumor volume was greater than 36cc, and ISBT was significantly better than the other two plans for tumor volumes larger than 80cc. A study by Kirisits et al. (4) showed that the 100% isodose of ICBT was at maximum 25 mm from the tandem axis at the level of point A, while the isodose could be moved to 31 mm when using IC/ISBT. Inspired by these reports, we divided all treatment plans into 4 groups based on parametrial disease extension widths of ≤3, 3.1–3.5, 3.6– 4, and >4cm because our results indicated that NMD was significant impacted by parametrial free needles. Compared with IC/ISBT, the dosimetric benefit of IC/IS+ISBT DP was only applicable to parametrial extension widths greater than 3 cm, which is consistent with the conclusion of Kirisits et al. (4). Moreover, the average NMD of all 77 IC/IS+ISBT DP treatment plans in our study could reach 3.64cm, but only 2.90cm after removing the free needles from the distal parametrium. In addition, the V200 of IC/IS+ISBT DP was lower than that of the IC/ISBT group. Although the dwell weight of the free needles was only 16.4%, it still reduced the dwell weight of the needles in the near center area. Therefore, we found it inappropriate to increase free needles in the parametrium just to increase the fitness of HR-CTV if the IC/ISBT could achieve the dosage requirements, thereby reducing unnecessary trauma by reducing the use of implanted needles. After all, higher V150, and V200 could provide better local control rate (13).

The American Brachytherapy Society recommends 80–90 Gy equivalent doses in 2 Gy fractions (EQD2) for D90 HR-CTV (14). Furthermore, it recommended at least 85 Gy for D90 HR-CTV if the tumor measured greater than 4cm after EBRT. The Vienna system study (15) pointed out that when the dose was increased from 81–90 Gy for large tumors, the local control rate increased from 71–90%. A recent study by Dang et al. (16) enrolled 100 cervical cancer patients treated with IC/ISBT. The 5-year LC, DMFS and OS reached 88.9, 81.8, and 77.9%, respectively. A prospective study (17) enrolled 33 patients with cervical cancer, given ICBT or IC/ISBT, the D90 of HR-CTV reached 88–92.9Gy, and the 1-year LC and OS were 84 and 91.3%, respectively. But the clinical results of IC/IS+ISBT DP have not been reported. In this study, patients received a median of 91.15GyEQD2 for D90 HR-CTV, which achieved the above dose range. Only 2 cases had local recurrence after 2 years of follow-up, showing an ideal local control rate. Univariate analysis showed that the parametrial extension width is a prognostic risk factor for PFS, indicating that the probability of treatment failure will be significantly increased when the parametrial extension width is greater than 3 cm. Therefore, it is particularly important to give sufficient doses to the distal parametrium. If possible, PET-CT should be used to accurately delineate the target volume for distal parametrium due to the major capability to identify high-risk RT areas (18). For similar patients, even more efforts to devise satisfactory individualized treatment plans will be necessary to address the difficult challenge of simultaneously increasing the dose for HR-CTV yet limiting the dose to OARs.

Previous studies have indicated limiting D2cc ≤ 90 Gy for the bladder and D2cc ≤ 70 Gy for the rectum, sigmoid and small bowel (19). The single dose limitation according to our treatment plan indicated a bladder D_2CC_ ≤ 5.88 Gy, and rectum and sigmoid D_2CC_ ≤ 4.98 Gy. In this study, the average dose of OARs were lower than the above-mentioned dose limit, and no grade 3 or 4 bladder LSE or rectum LSE were observed after 2 years follow-up. All the OARs in the 77 IC/IS+ISBT DP plans enrolled in this study were below the above dose limits. The D_2CC_ for rectum and sigmoid for IC/IS+ISBT DP showed little advantage to IC/ISBT when the parametrial extension widths were less than 3cm. However, the addition of free needles in the distal parametrium significantly contributed to the dose limitation of the bladder, rectum and sigmoid when the parametrial extension widths exceeded 3cm, especially for the protection of the rectum. In order to cover the area of the distal parametrium as much as possible, the IC/ISBT treatment plan could only increase the dose of the channels closest to the distal parametrium, inevitably leading to an increase in the thickness of the 100% isodose curve. Therefore, IC/IS+ISBT DP lowered the risk of side effects. In addition, because of the invasive nature of this operation, complications could occur during implantation such as bleeding and infection. The incidence of infections observed in this study was judged acceptable; bleeding due to IS needles was minimal, controlled just with vaginal tamponade. Overall, the patients tolerated IC/IS+ISBT DP well.

At present, ICBT combined with EBRT with parametrial boosting or interstitial brachytherapy (ISBT) have been used by some departments for locally advanced cervical cancer. In order to compare the dose distribution of IC/ISBT and ICBT+EBRT, Mohamed et al. (20) enrolled 51 patients with locally advanced cervical cancer from 2008 to 2011. The results showed that the HR-CTV D90 provided by the IC/ISBT group was significantly higher than that of the ICBT+EBRT group (the HR-CTV D90 of 3 cases in the ICBT+EBRT group was less than 79 Gy, while all patients in the IC/ISBT group were higher than 84 Gy). Additionally, the IC/ISBT group also had obvious advantages regarding D_2CC_ of OARs. Furthermore, EBRT could not be synthesized with brachytherapy perfectly, leading to omission and overlap of doses, and inaccurate dose assessment. Lindegaard et al. (21) also pointed out that parametrial boosting by EBRT could only provide doses to the HR-CTV and IR-CTV selectively, with consequent significantly increased radiation dose to OARs. The conclusion: for cervical cancer with associated parametrial disease extension, EBRT supplementation cannot replace brachytherapy. ISBT, either by freehand or template implantation, is a preferred treatment option for locally advanced cervical cancer. The angle and position of the implant needles were unlimited, and it could provide excellent DVH parameters (22). However, Hsu et al. (23) pointed out that although ISBT has a high degree of conformity to the HR-CTV, the treatment volume receiving >180% of the prescribed dose was significantly lower than ICBT (17 vs 31cc). Although there are no direct comparison reports, previous studies have shown that the size of the central high-dose area could affect the local control rate (13). In addition, the precisely accurate location and reproducibility of ISBT with a free hand technique was limited. IC/IS+ISBT DP minimizes the use of free needles and improves accuracy and reproducibility. Takahiro et al. (24) compared dosimetric differences between IC/ISBT and ISBT, and the results showed that the V100 of IC/ISBT to be minimally different from ISBT. Therefore, we contend that the results of this and other studies demonstrate that IC/IS+ISBT DP has advantages in dosimetry and treatment accuracy for cervical cancer complicated by distal parametrial disease extension compared with ISBT.

In summary, the evidence indicates that the radiotherapy technique of IC/IS+ISBT DP has excellent dosimetry parameters for cervical cancer patients with distal parametrial extension (particularly parametrial extension widths greater than 3cm). However, although brachytherapy appears effective and valuable, especially in gynecological malignancies, and cannot be replaced by intensity modulated or stereotactic radiotherapy, it has been progressive decline over the past decades. And the development of brachytherapy technology is relatively backward compared with external beam radiotherapy. It is necessary to strengthen the clinical practice, education, research and communication (25). Practical education should be given special attention due to the higher technical requirements for the operator, especially the technology mentioned in our research. It is recommended to set up more brachytherapy training institutions, aiming to educate physicians/medical physicists in brachytherapy through a multidisciplinary approach such as GEMELLI-INTERACTS (Interventional Radiotherapy Active Teaching School) mode (26), to better serve patients.

## Data Availability Statement

The raw data supporting the conclusions of this article will be made available by the authors, without undue reservation.

## Ethics Statement

Written informed consent was obtained from the individual(s) for the publication of any potentially identifiable images or data included in this article.

## Author Contributions

H-DQ performed the statistical analysis and drafted the manuscript. G-HC participated in the design of this study and performed manuscript review. D-MH and NZ performed patients follow-up and data collection. ZM devised the treatment plan. All authors contributed to the article and approved the submitted version.

## Funding

This work was partially supported by grants from the National Natural Science Foundation of China [grant numbers 82073331, 81201737, 31600679,81703034]; Project of Science and Technology Department of Jilin Province (grant number 20190303151SF);Horizontal Project of Jilin University [grant numbers 2019YX435, 2019155].

## Conflict of Interest

The authors declare that the research was conducted in the absence of any commercial or financial relationships that could be construed as a potential conflict of interest.
